# Molecular Evolutionary Dynamics of Coxsackievirus A6 Causing Hand, Foot, and Mouth Disease From 2021 to 2023 in China: Genomic Epidemiology Study

**DOI:** 10.2196/59604

**Published:** 2024-07-31

**Authors:** Yu Chen, Shouhang Chen, Yuanfang Shen, Zhi Li, Xiaolong Li, Yaodong Zhang, Xiaolong Zhang, Fang Wang, Yuefei Jin

**Affiliations:** 1 Department of Infectious Diseases, Children’s Hospital Affiliated to Zhengzhou University, Zhengzhou, China; 2 College of Public Health, Zhengzhou University, Zhengzhou, China; 3 Henan International Joint Laboratory of Children’s Infectious Diseases, Children's Hospital Affiliated to Zhengzhou University, Zhengzhou, China; 4 NHC Key Laboratory of Birth Defects Prevention, Henan Key Laboratory of Population Defects Prevention, Zhengzhou, China

**Keywords:** coxsackievirus A6, hand, foot, and mouth disease, evolution, molecular epidemiology, China, CV-A6, HFMD

## Abstract

**Background:**

Hand, foot, and mouth disease (HFMD) is a global public health concern, notably within the Asia-Pacific region. Recently, the primary pathogen causing HFMD outbreaks across numerous countries, including China, is coxsackievirus (CV) A6, one of the most prevalent enteroviruses in the world. It is a new variant that has undergone genetic recombination and evolution, which might not only induce modifications in the clinical manifestations of HFMD but also heighten its pathogenicity because of nucleotide mutation accumulation.

**Objective:**

The study assessed the epidemiological characteristics of HFMD in China and characterized the molecular epidemiology of the major pathogen (CV-A6) causing HFMD. We attempted to establish the association between disease progression and viral genetic evolution through a molecular epidemiological study.

**Methods:**

Surveillance data from the Chinese Center for Disease Control and Prevention from 2021 to 2023 were used to analyze the epidemiological seasons and peaks of HFMD in Henan, China, and capture the results of HFMD pathogen typing. We analyzed the evolutionary characteristics of all full-length CV-A6 sequences in the NCBI database and the isolated sequences in Henan. To characterize the molecular evolution of CV-A6, time-scaled tree and historical population dynamics regarding CV-A6 sequences were estimated. Additionally, we analyzed the isolated strains for mutated or missing amino acid sites compared to the prototype CV-A6 strain.

**Results:**

The 2021-2023 epidemic seasons for HFMD in Henan usually lasted from June to August, with peaks around June and July. The monthly case reporting rate during the peak period ranged from 20.7% (4854/23,440) to 35% (12,135/34,706) of the total annual number of cases. Analysis of the pathogen composition of 2850 laboratory-confirmed cases identified 8 enterovirus serotypes, among which CV-A6 accounted for the highest proportion (652/2850, 22.88%). CV-A6 emerged as the major pathogen for HFMD in 2022 (203/732, 27.73%) and 2023 (262/708, 37.01%). We analyzed all CV-A6 full-length sequences in the NCBI database and the evolutionary features of viruses isolated in Henan. In China, the D3 subtype gradually appeared from 2011, and by 2019, all CV-A6 virus strains belonged to the D3 subtype. The VP1 sequences analyzed in Henan showed that its subtypes were consistent with the national subtypes. Furthermore, we analyzed the molecular evolutionary features of CV-A6 using Bayesian phylogeny and found that the most recent common ancestor of CV-A6 D3 dates back to 2006 in China, earlier than the 2011 HFMD outbreak. Moreover, the strains isolated in 2023 had mutations at several amino acid sites compared to the original strain.

**Conclusions:**

The CV-A6 virus may have been introduced and circulating covertly within China prior to the large-scale HFMD outbreak. Our laboratory testing data confirmed the fluctuation and periodic patterns of CV-A6 prevalence. Our study provides valuable insights into understanding the evolutionary dynamics of CV-A6.

## Introduction

Hand, foot, and mouth disease (HFMD) is an acute infectious disease that mainly affects children aged <5 years; its symptoms include fever and rashes on the hands, feet, oral mucosa, and buttocks [1]. In China, the annual peak season for HFMD outbreaks usually lasts from April to June. Some areas also experience a surge of HFMD from October to November, notably in Southern China [2,3]. HFMD has increasingly become a public health concern and has a substantial effect on the disease burden and economic status of the affected regions [4,5]. HFMD is primarily caused by enterovirus (EV) A, and the most common pathogens are EV-A71 and coxsackievirus (CV) A16 [6]. Recently, CV-A6 has gained global concern, with a large number of patients reported in Finland [7], Singapore [8], Japan [9], Spain [10], and Thailand [11]. Since the end of 2012, CV-A6 has emerged as the main pathogen of HFMD in China [12]. By 2013, Guangdong, Jilin, and Beijing had recorded cases of HFMD caused by CV-A6 [13-15]. However, most studies have focused on major serotypes such as EV-A71 and CV-A16, with a limited amount of information on other EV serotypes. This is largely due to these other EV serotypes generally causing lesser disseminated case. CV-A6 is one of the most prevalent EVs in the world in recent years, and it is a new variant that has undergone genetic recombination and evolution. It is specifically associated with genotype D [16]. The CV-A6 D3 subtype associated with HFMD was first described in 2008 following an outbreak in Finland and subsequently spread to other European countries [16,17]. After 2018, the D3 subtype emerged in Asia and was described from samples collected during outbreaks in Philippines [18], Vietnam [19], and other countries [20]. HFMD cases caused by CV-A6 tend to have clinical characteristics that differ from those of typical HFMD, such as atypical herpes [21], and can lead to severe neurological or cardiopulmonary complications, such as encephalitis, aseptic meningitis, encephalomyelitis, acute flaccid paralysis, and fatal cardiopulmonary complications [22-24]. Recombination and evolution could potentially alter not only the clinical manifestations of HFMD but also heighten the disease’s pathogenicity due to cumulative nucleotide mutations [25]. Most importantly, CV-A6 appears to influence a more extensive demographic of children, inducing a more severe and prolonged ailment compared to other EVs [26]. However, the evolution and transmission of CV-A6 in China have been relatively underexplored in recent years [27]. In this study, 34 HFMD-associated CV-A6 strains were isolated from Henan Children’s Hospital and the Third Affiliated Hospital of Zhengzhou University, which greatly enriched the global CV-A6 VP1 sequence database. We downloaded all CV-A6 full-length sequences before January 2024 from the NCBI database to characterize the evolution of the isolated CV-A6 strains and to analyze the temporal and geographic distribution of different CV-A6 subtypes using Bayesian Markov chain Monte Carlo (MCMC) methodology in China. Furthermore, this study characterized the amino acid mutations of the VP1 gene. This study aimed to provide insight into the molecular epidemiology of CV-A6 and establish a link between disease development and viral evolution.

## Methods

### Collection of Epidemiological Data

HFMD is classified as a legally reportable infectious disease in China. Standardized case questionnaires are used to collect demographic and epidemiologic data, which are reported in a timely manner to the web-based China Disease Prevention and Control Information System [2,28]. For the purposes of this study, these aforementioned data were extracted from this national database. In our analysis, an “epidemic season” for HFMD means that the number of cases reported in that month constituted at least 15% of the total cases reported throughout the year. The “epidemic peak” was identified as the month witnessing the highest influx of reported cases.

### Data Sources

The baseline data of HFMD cases in Henan Children’s Hospital (Children’s Hospital Affiliated to Zhengzhou University) and the Third Affiliated Hospital of Zhengzhou University (Henan Maternal and Child Health Hospital of Henan Province), which had advantages in the diagnosis and treatment of HFMD, were also included in this study. From both hospitals, the pathogenetic data of HFMD cases from 2021 to 2023 were selected as representation. Biological samples including fecal samples and throat swabs were collected from HFMD cases for the study.

### Ethical Consideration

This study received ethical approval from the Committee for Ethical Review of Zhengzhou University (ZZUIRB2023-180), and written informed consent was obtained from all participants’ parents. The obtained data have been anonymized or deidentified.

### RNA Extraction

Viral RNA was extracted from fecal samples of the abovementioned HFMD cases. Each fecal sample was stored at −80 °C; the sample was thawed at room temperature, and 0.1 g of the sample was placed in a 1.5-mL sterile, enzyme-free, grinding tube. Next, 1 mL of TRIzol reagent (Thermo Fisher Scientific) along with 2 sterile, enzyme-free, steel beads (3 mm) were added to the tube. The tube was then placed in a precooled tissue grinder and grinded 3 times for 30 seconds, each time at a frequency of 60 Hz. Chloroform was then added and the mixture was centrifuged, retaining the supernatant. Isopropanol was then added and allowed to stand for 10 minutes before being placed in the centrifuge again and discarding the supernatant. Then, 1 mL of 75% ethanol was added and mixed thoroughly, and another centrifugation was initiated. At the final stage, the supernatant was discarded, and the precipitate was dried and resuspended in 30 μL of RNase-free water. The concentration of RNA was measured using a NanoDrop spectrophotometer (Thermo Fisher Scientific).

### Viral Sequencing

Viral RNA was converted to complementary DNA using the Hifair II First Strand cDNA Synthesis Kit (Yeasen Biotechnology) according to the instructions. The VP1 section of the CV-A6 sequence was amplified using primers (forward primer: AAGGACACTGACGAGATTCAGC; reverse primer: AGTGGCGAGATGTCGGTTT; 1026 base pairs). Then, the amplified fragments were sent to Beijing Liuhe Huada Gene Technology Co., Ltd. for sequencing.

### Data Set Construction

For the study, we retrieved the full-length sequence of CV-A6 from the GenBank database using “Coxsackievirus A6” as an index term with a cutoff date of January 1, 2024. From the GenBank database, all CV-A6 (near) whole-genome sequences (sequence length of 6600-7500 base pairs) were extracted. Any disreputable or subpar sequences were excluded. This includes laboratory-adapted strains, clones, strains with elevated passage numbers, sequences containing numerous unascertained bases, and mislabeled sequences that belonged to other serotypes. Ultimately, 858 near full-length sequences were gathered from GenBank. We constructed a phylogenetic evolutionary tree using the full-length CV-A6 sequences of 858 different strains to understand the phylogeny of CV-A6 strains in China. All data sets are listed in Multimedia Appendix 1. Next, we selected representative strains from different countries and years to understand the phylogeny based on the CV-A6 VP1 isolates and excluded inferior-quality sequences. The representative strains should cover more provinces of China to ensure a high geographic representation and cover each cluster of the phylogenetic trees to acquire a high phylogenic representation. After strict quality control, we compiled a data set of 313 VP1 sequences for phylogenetic and phylodynamic analyses. The samples cover 16 countries worldwide, and the time series were from 1970 to 2023. All data sets are listed in Multimedia Appendix 2. The complete VP1 nucleotide sequences of CV-A6 was 915 nucleotides long, which encode 305 amino acids.

### Phylodynamic Analysis

We used MEGA 7.0 software [29] to compare the VP1 nucleotide sequence of CV-A6. Then, we calculated a maximum likelihood (ML) phylogenetic tree using IQ-TREE (version 1.6.12) [30] under the best-fit nucleotide substitution model as designated by Model Finder. For CV-A6, the SYM+G4 model was deemed the best fit, as outlined in Table 1 [31]. We used the ultrafast bootstrap method with 1000 replicates for our analyses. Analysis of temporal molecular evolutionary signals for the data set was conducted using TempEst (version 1.5) [32]. In brief, regression analyses were used to determine the relationship between sampling dates and root-to-tip genetic divergence obtained from the ML phylogeny. The slope of the regression line provides an estimate of the rate of evolution in substitutions per site per year, and the intercept with the time-axis constitutes an estimate of the age of the root. The time-scaled tree along with historical population dynamics were estimated using the BEAST package (version 1.10.4) [33]. Information regarding the time of the most recent common ancestor (tMRCA) and the rate of evolution (represented as substitutions per site per year) were derived from this estimation. To reconstruct the phylogeny, we used a Bayesian MCMC approach [33]. The nucleotide substitution model was tested through the use of Model Finder, with the Bayesian Sky grid model featuring a relaxed molecular clock being used. The MCMC lengths of 50,000,000 generations were completed with individual samplings convened every 5000 generations. Output from BEAST was analyzed within the Tracer program (version 1.7.1) [34] to ensure convergence through graphical checks and adequate quality control parameters of the posterior distribution (effective sample size>200). The maximum clade credibility (MCC) tree was ascertained through the use of Tree Annotator (version 1.10.4) and was depicted in FigTree (version 1.4.4) [35] in Multimedia Appendix 3. Then, the tMRCA and its 95% highest posterior density (HPD) were quantified in chronological years.

**Table 1.** The best-fit nucleotide substitution model of coxsackievirus A6 determined by Model Finder.

No.	Model	Logarithm of site likelihood	*df*	AIC^a^	AICc^b^	BIC^c^
1	JC	19,581.406	149	39,460.812	39,488.82	39,460.812
2	JC+G4	18,154.721	150	36,609.441	36,637.842	37,429.203
3	TN+F	18,121.2	154	36,550.399	36,580.405	36,580.405
4	TN+F+G4	16,528.704	155	33,367.408	33,397.823	34,214.496
5	TNe	18,120.71	151	36,543.421	36,572.219	37,368.648
6	TNe+G4	16,535.95	152	33,375.899	33,405.097	34,206.592
7	K2P	18,178.311	150	36,656.621	36,685.022	37,476.383
8	K2P+G4	16,555.576	151	33,413.152	33,441.95	34,238.38
9	K2P	18,178.311	150	36,656.621	36,685.022	37,476.383
10	K2P+G4	16,555.576	151	33,413.152	33,441.95	34,238.38
11	F81+F	19,582.947	152	39,469.893	39,499.091	40,300.586
12	F81+F+G4	18,149.835	153	36,605.671	36,635.271	37,441.828
13	HKY+F	18,166.577	153	36,639.155	36,668.755	37,475.312
14	HKY+F+G4	16,544.798	154	33,397.597	33,427.603	34,239.219
15	SYM	18,096.173	154	36,500.345	36,530.352	37,341.968
16	SYM+G4	16,515.599	155	33,341.197	33,371.612	34,188.285
17	TIM+F	18,119.787	155	36,549.575	36,579.99	37,396.663
18	TIM+F+G4	16,528.381	156	33,368.762	33,399.589	34,221.315
19	TVM+F	18,132.147	156	36,576.294	36,607.121	37,428.847
20	TVM+F+G4	16,512.717	157	33,339.435	33,370.676	34,197.453
21	TVMe	18,153.435	153	36,612.869	36,642.47	37,449.027
22	TVMe+G4	16,536.233	154	33,380.465	33,410.472	34,222.088
23	GTR+F	18,086.981	157	36,487.962	36,487.962	37,345.98
24	GTR+F+G4^d^	16,495.349	158	33,306.698^e^	33,338.357^e^	34,170.181^e^

a AIC: Akaike information criterion.

b AICc: corrected Akaike information criterion.

c BIC: Bayesian information criterion.

d Best-fit model: GTR+F+G4 was chosen according to BIC.

e Best fit.

### Base Substitution and Amino Acid Mutation Analysis

This study further analyzed the differences between the 34 CV-A6 strains and the prototype CV-A6 strain. The nucleotide sequences and deduced amino acid sequences of the above 34 strains were compared with the amino acid sequence of the CV-A6 prototype strain. Amino acid sites with mutations or deletions were analyzed using BioEdit software (version 7.2.5.0) [36].

## Results

### The Epidemiology and Etiology of HFMD in Henan, China, from 2021 to 2023

**Figure 1.**
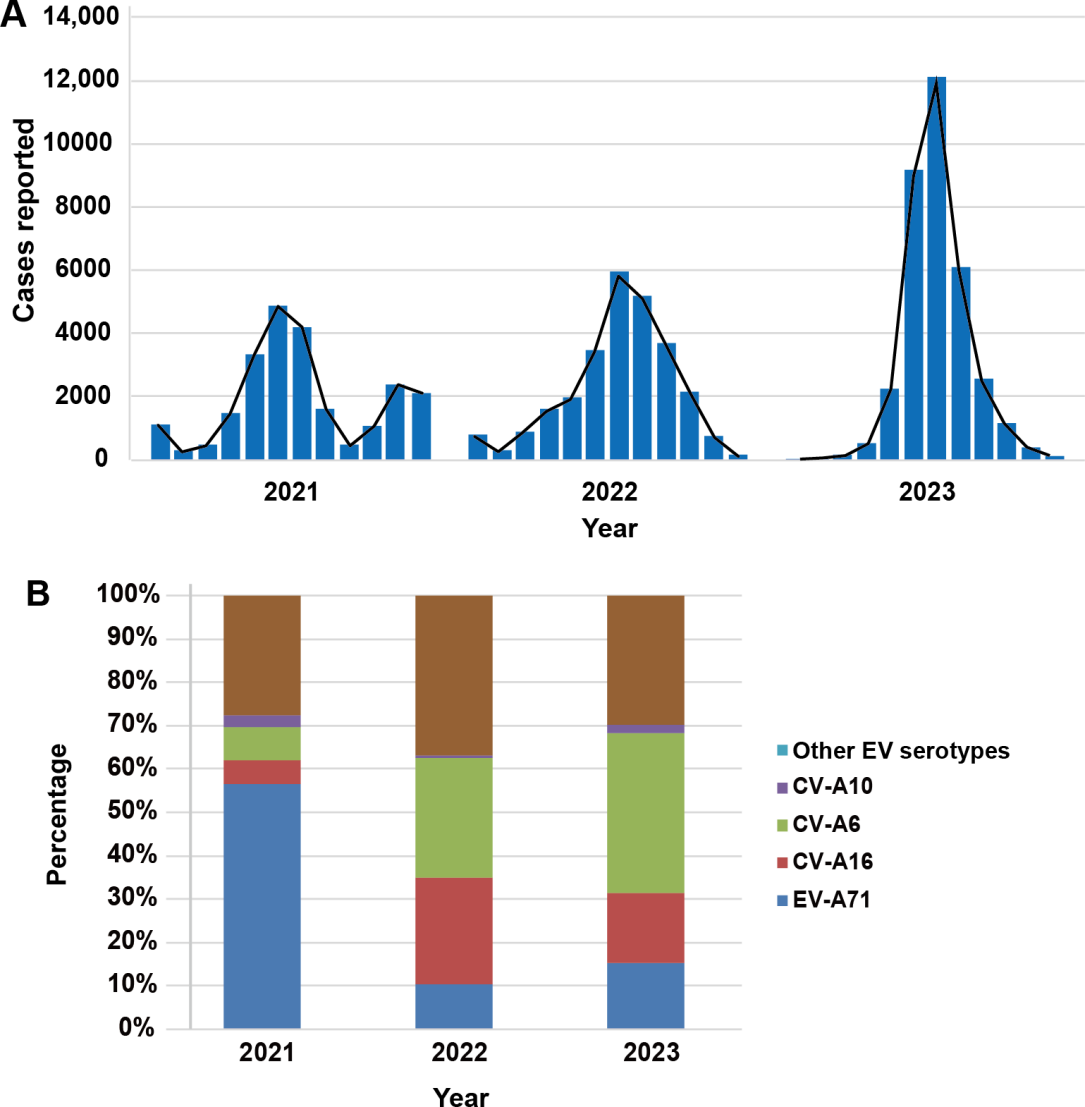
Pathogen spectrum of HFMD in Henan, China, from 2021 to 2023: (A) the monthly incidence of HFMD and (B) composition ratio of various EVs in Henan, from 2021 to 2023. CV: coxsackievirus; EV: enterovirus; HFMD: hand, foot, and mouth disease.

From 2021 to 2023, a total of 85,060 HFMD cases were included in our study (n=23,440, 27.56% in 2021; n=26,914, 31.64% in 2022; and n=34,706, 40.8% in 2023). Our data showed that the epidemic season in Henan usually lasted from June to August, with peaks around June and July (Figure 1A). During this period, the monthly case reporting rate ranged from 20.7%(4854/23,440) to 35% (12,135/34,706) of the total annual number of cases. Next, we analyzed the pathogen composition of 2850 laboratory-confirmed cases from Henan Children’s Hospital and the Third Affiliated Hospital of Zhengzhou University. The analysis identified 8 EV types, but 1145 EV-positive specimens (40.84%, 1164/2850) could not be typed. Among them, CV-A6 was the most frequently identified EV type, accounting for 652 (22.88%) out of 2850 cases, followed by CV-A16 (n=624, 21.89%), EV-A71 (n=326, 11.44%), and CV-A10 (n=84, 2.95%). In 2021, CV-A16 was the main pathogen of HFMD with 23.48% (331/1410) of the cases. CV-A6 emerged as the primary pathogen for HFMD in 2022 and 2023, constituting 27.73% (203/732) and 37.01% (262/708) of cases, respectively. Most importantly, the detection rate of CV-A6 spiked from 13.26% (187/1410) in 2021 to 37.01% (262/708) in 2023 (Table 2). Out of all identified EV pathogen types, CV-A6 had the highest composition ratio at 22.88% (652/2850), and the composition ratios of other pathogen types are depicted in Figure 1B.

**Table 2.** Enterovirus (EV) serotypes of patients with hand, foot, and mouth disease (n=2850) in Henan, China, from 2021 to 2023.

Year	EV-A71, n (%)	CV^a^-A16, n (%)	CV-A6, n (%)	CV-A10, n (%)	Other EV serotypes, n (%)	Total, n (%)
2021 (n=1410)	141 (10)	331 (23.48)	187 (13.26)	67 (4.75)	684 (48.51)	1410 (100)
2022 (n=732)	76 (10.38)	180 (24.59)	203 (27.73)	4 (0.55)	269 (36.75)	732 (100)
2023 (n=708)	109 (15.40)	113 (15.96)	262 (37.01)	13 (1.83)	211 (29.80)	708 (100)
Total (n=2850)	326 (11.44)	624 (21.89)	652 (22.88)	84 (2.95)	1164 (40.84)	2850 (100)

a CV: coxsackievirus.

### Subtyping Analysis of CV-A6

**Figure 2.**
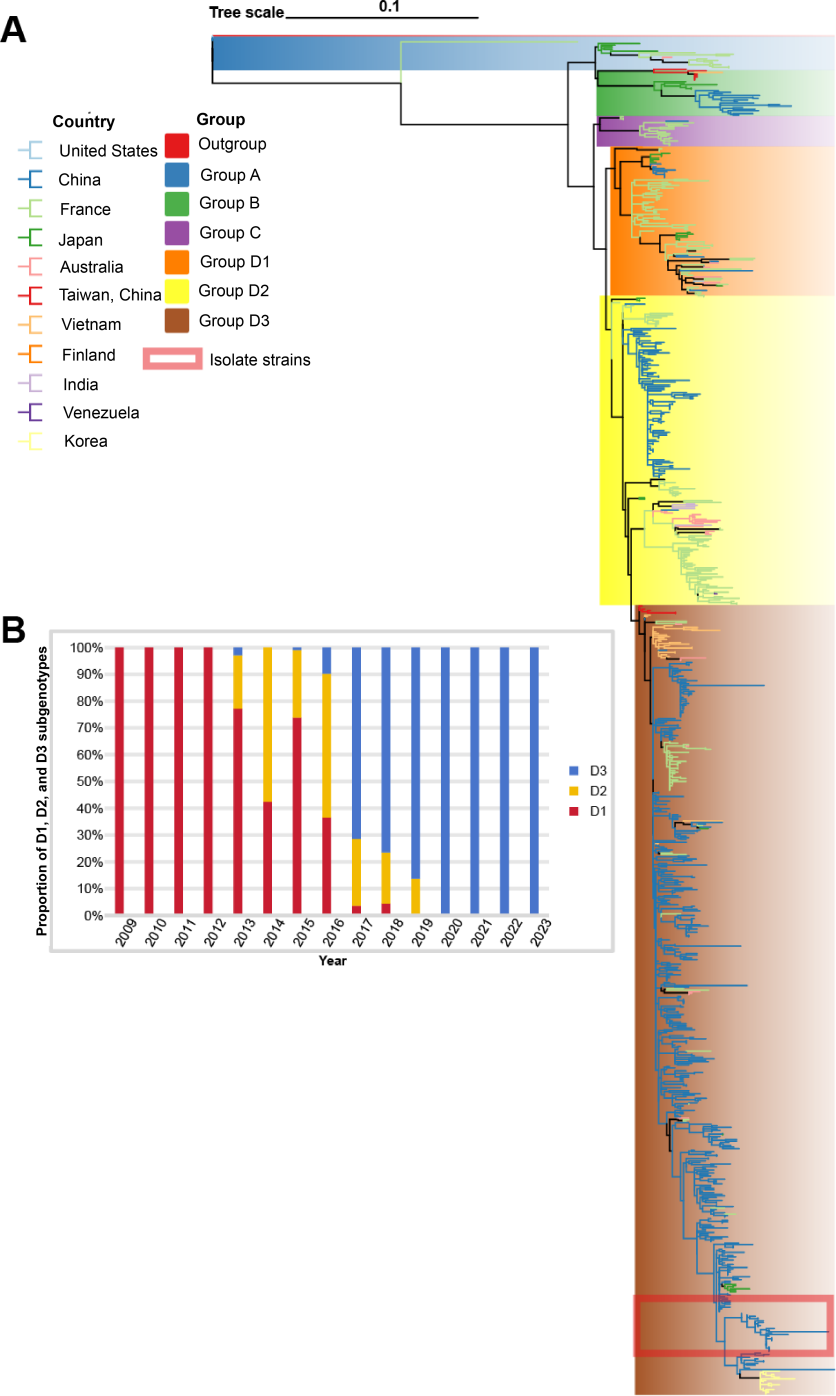
Subtyping analysis of CV-A6. (A) Genetic evolutionary characteristics of enteroviruses isolated in Henan, China. Maximum likelihood phylogenetic trees for enteroviruses were constructed with CV-A6 full-length sequences. The red box marked the strains that have been isolated this study. The colors of the branches represent countries, and the colors on the strains represent subtypes. (B) Annual percentage distribution of subgenotypes D1, D2, and D3 associated with HFMD from 2009 to 2023 in mainland China. CV: coxsackievirus; HFMD: hand, foot, and mouth disease.

To analyze the genomic characteristics and patterns of genetic evolution of isolated strains, we downloaded the full-length sequence of CV-A6 from the NCBI database and performed phylogenetic analysis on the strains isolated in this study. Genogroups A, B, and C contained very few members due to the lack of complete VP1 nucleotide sequence data and limited virus circulation. The virulent strain isolated in this study was subtype D3 (Figure 2A). In this study, the full-length sequences in China were analyzed by subgenotype and year. It was found that since 2013, the prevalence of subgenotype D1 viruses had gradually declined, and a large number of D2 strains began to appear, accompanied by a small number of D3 subtypes. Since 2017, the D3 subtype has begun to proliferate, and by 2020, all CV-A6 strains in China were of the D3 subtype (Figure 2B).

**Figure 3.**
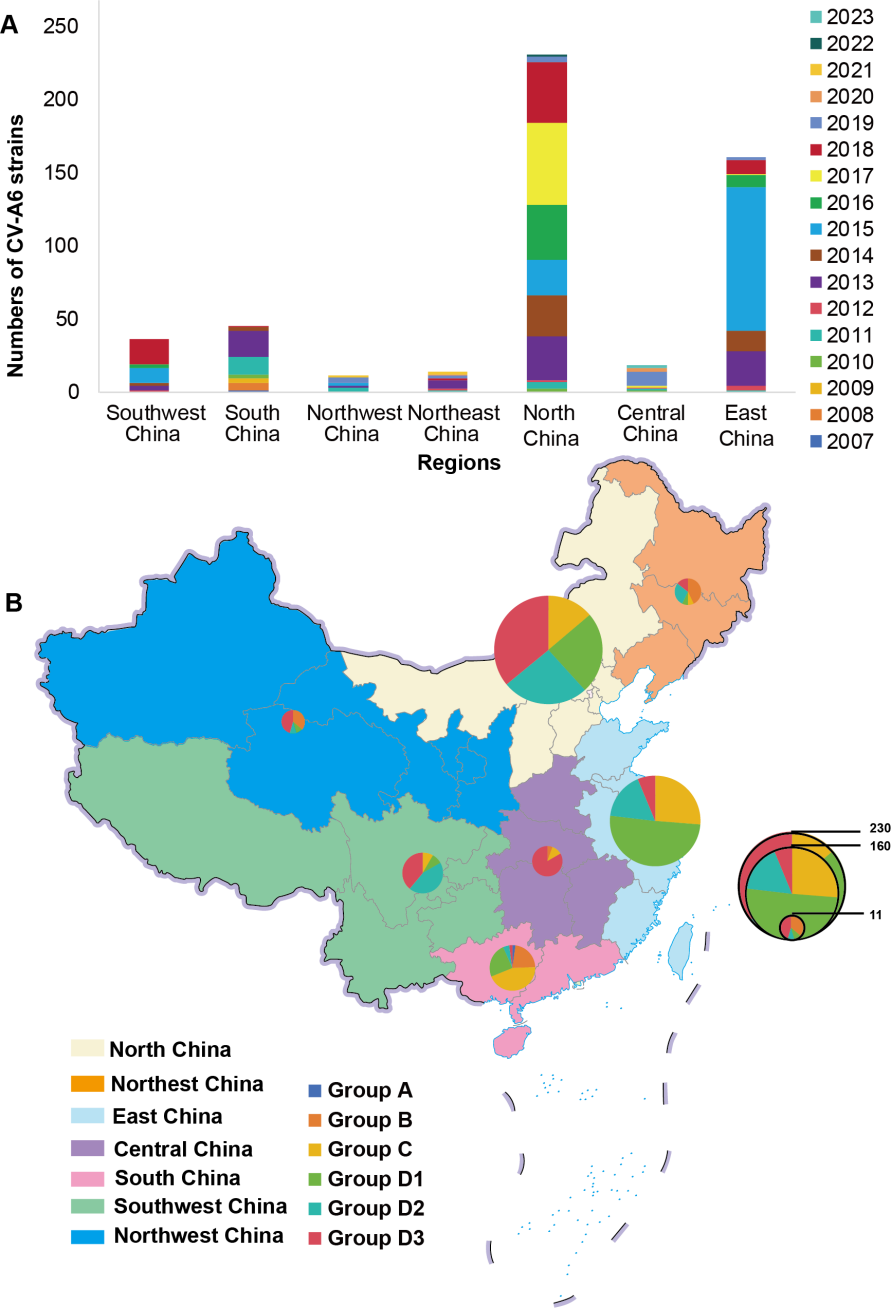
The temporal and geographical distribution characteristics of CV-A6 in China. (A) Annual and regional distribution of 514 CV-A6 strains in China. (B) The distribution of CV-A6 strains associated to HFMD in 7 significant regions of China, where both region and sequence quantity are denoted and differentiated by the provided color key. The colors on the map represent different geographical areas, and the colors on the pie charts represent the pathogens of HFMD. CV: coxsackievirus; HFMD: hand, foot, and mouth disease.

In this study, a total of 514 full-length CV-A6 sequences from various regions within China were downloaded from the GenBank database. Only 1 CV-A6 isolate was obtained before 2008, and 44 isolates were reported between 2008 and 2012; strains isolated between 2013 and 2023 accounted for 91.24% (469/514; Figure 3A). These 514 isolates provide a representative coverage of 7 different Chinese geographic regions: East China (comprising Shandong, Jiangsu, Zhejiang, Fujian, Anhui, Shanghai, and Taiwan), South China (Guangdong, Guangxi, Hainan, Hong Kong, and Macao), Central China (Henan, Hubei, Hunan, and Jiangxi), North China (Inner Mongolia, Tianjin, Beijing, Hebei, and Shanxi), Northwest China (Xinjiang, Qinghai, Shaanxi, Ningxia, and Gansu), Southwest China (Sichuan, Yunnan, Guizhou, and Chongqing), and Northeast China (Heilongjiang, Jilin, and Liaoning; Figure 3B).

### Molecular Evolution Analysis of CV-A6

**Figure 4.**
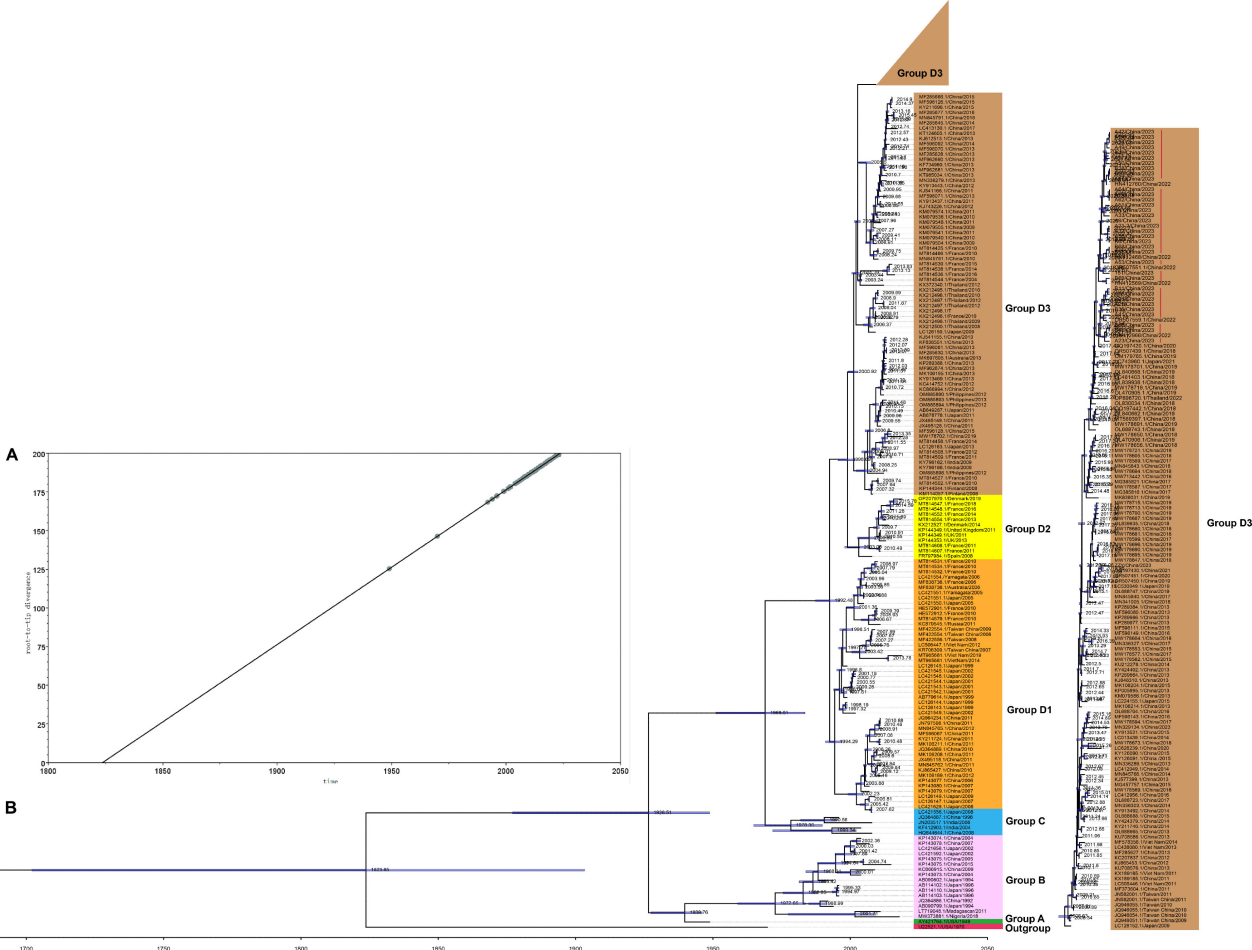
Molecular evolution analysis of CV-A6. (A) Temporal signal analysis of root-to-tip divergence regression versus date (*R*^2^=1). (B) The phylogenetic tree was constructed with maximum clade credibility for 313 VP1 sequences of CV-A6 strains. The purple bars at the nodes indicate the 95% HPDs of tMRCAs. Red vertical lines represent the 34 CV-A6 strains isolated in this study. CV: coxsackievirus; HPD: highest posterior density; tMRCA: time of the most recent common ancestor.

To gain insights into the evolutionary patterns of the isolated CV-A6 strains in our study and their relationships with previous CV-A6 strains and other viruses from different regions, we assembled a data set comprising 313 VP1 coding sequences. Using this data set, we constructed an initial ML tree for root-to-tip analysis. This analysis revealed a strong positive temporal signal with an *R*^2^ value of 1, indicating a clear evolutionary trend over time (Figure 4A). We developed an MCC tree that estimated the divergence times of CV-A6 based on the 313 full-length VP1 sequences. The MCC tree exhibited consistency with the phylogenetic tree in terms of topology and typing results (Figure 4B). Furthermore, the mean substitution rate and tMRCA were generally calculated by temporal phylogenies for the evolution of CV-A6 full-length VP1 sequences. The results indicated a mean substitution rate of 4.5456×10^−3^ (95% HPD 3.8466×10^−3^ to 5.3796×10^−3^) substitution per site per year. The predicted tMRCA for the EV-A71 prototype strain was approximately 1824 (95% HPD 1702-1902). The ancestor of global CV-A6 isolates emerged approximately 108 years ago, dating the origin of CV-A6 to around 1915 (95% HPD 1870-1948). The specific details of the tMRCA of the genetic subtypes (A-D) of the CV-A6 strains, along with their respective 95% HPD ranges, are shown in Table 3. Furthermore, the earliest estimated time of CV-A6 transmission in China, as determined through BEAST analysis, dates back to 1989 (95% HPD 985-1991). This estimation aligns with the node date of the CV-A6 Chinese cluster as displayed in the MCC tree. In addition, our analysis predicted that the tMRCA for the isolated CV-A6 strains could trace back to 2019 (95% HPD 2018-2020).

**Table 3.** The mean and 95% HPD^a^ values of the tMRCA^b^ and evolutionary rate of coxsackievirus A6.

Genetic subtypes	tMRCA (year), mean (95% HPD)		Evolutionary rate (×10^–3^ substitutions/site/year), mean (95% HPD)	
Group A	1940 (1929‐1951)		3.31 (0.24‐11.66)	
Group B	1973 (1959‐1984)		2.39 (0.46‐7.7)	
Group C	1978 (1971‐2003)		2.81 (0.27‐10.11)	
Group D1	1994 (1992‐1997)		2.47 (0.15‐8.53)	
Group D2	2003 (1998‐2007)		6.46 (0.98‐3.18)	
Group D3	2000 (1999‐2003)		5.30 (0.80‐15.94)	

a HPD: highest posterior density.

b tMRCA: time of the most recent common ancestor.

### Amino Acid Variation Site Analysis of CV-A6

**Figure 5.**
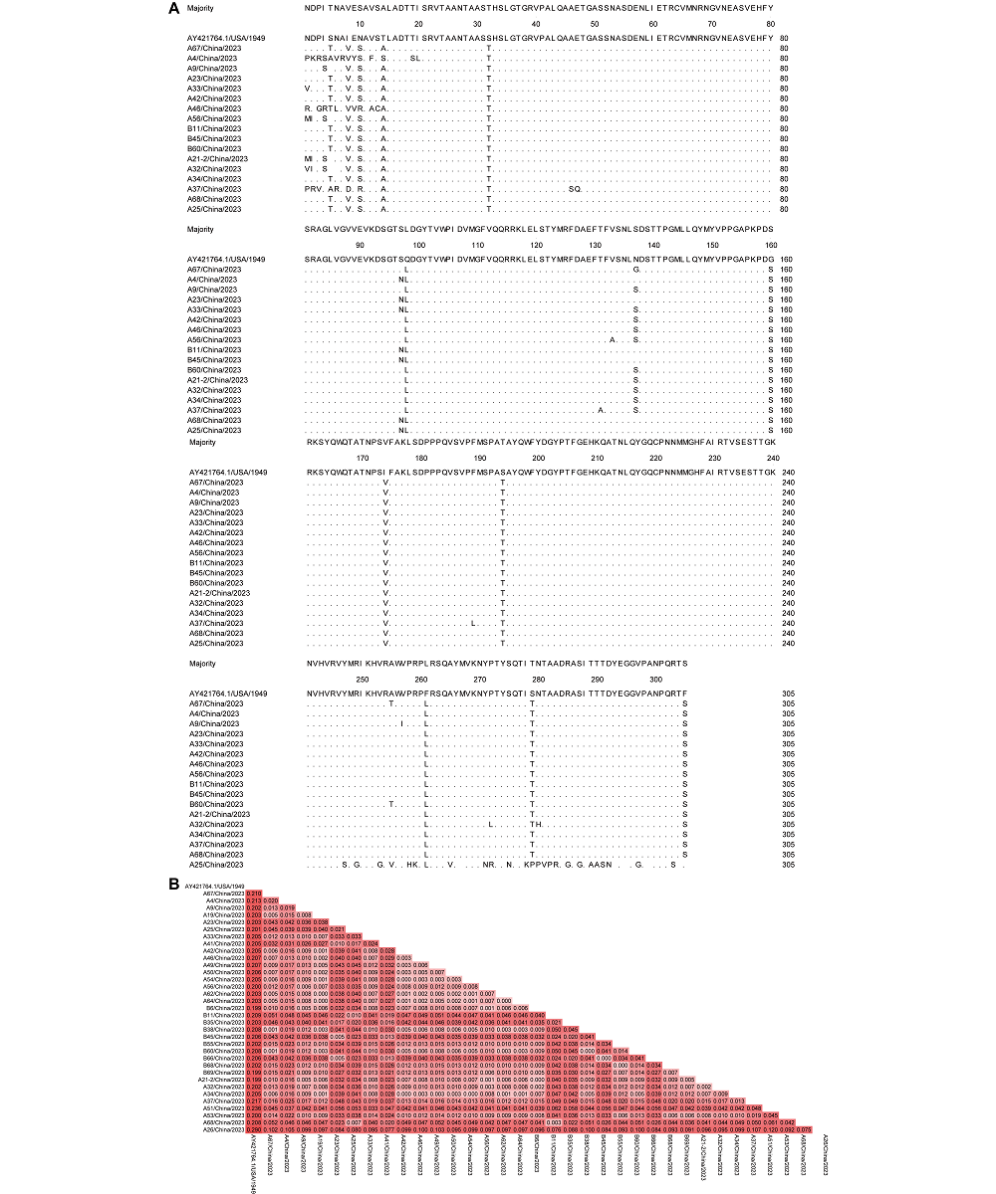
Amino acid mutation sites and genetic differences analysis of the VP1 gene. (A) Analysis of amino acid mutations in the VP1 gene of the isolated strains of CV-A6. (B) Estimates of evolutionary divergence between sequences of isolated CV-A6 strains and the prototype CV-A6 strain (AY421764.1). Values are indicated by color shading. Analyses were conducted using the maximum composite likelihood model. The analysis involved 35 nucleotide sequences. CV: coxsackievirus.

The 305 amino acids encoded in the VP1 gene of 34 CV-A6 isolates were compared with Gdula (AY421764.1) using MegAlign software (DNASTAR) to determine the amino acid mutation levels. In this study, we excluded strains with identical amino acid mutation sites and retained 17 strains with different mutation sites (Figure 5A). Amino acid substitutions or deletions occurred at all 11 of these sites (loci 5, 8, 10, 14, 32, 98, 160, 174, 194, 261, and 279). In addition, some strains had amino acid substitutions at locus 137. Unresolved questions about CV-A6 mutations need to be investigated to understand their roles in CV-A6 evolution and to enrich the information on relevant mutations in EVs. Further monitoring and analysis of mutated loci and epidemiological investigations are needed to determine whether mutations occurring at these loci are associated with changes in virus transmission, pathogenicity, and virulence. From the analysis of evolutionary differences between the CV-A6 strains isolated in this study and the prototype CV-A6 strain, we found that the genetic differences between the isolates were small, while the differences between the isolates and the prototype strain (AY421764.1) were large, and the values were all greater than 0.199 (Figure 5B). The greater the genetic distance, the higher the probability of mutation and recombination in the virus and the CV-A6 virus evolving over time.

## Discussion

HFMD outbreaks have proven to be a substantial public health concern in the Asia-Pacific region, particularly in China, over the past decade [2,37]. Consequently, attention from researchers toward CV-A6 has been growing. The sporadic new cases of CV-A6 infections over the past decade have indicated a possible worldwide resurgence of EV pathogens [7,17,38,39]. In this study, the epidemic season in Henan usually lasted from June to August, with peaks around June and July, from 2021 to 2023. Next, we analyzed the pathogen composition of 2850 laboratory-confirmed cases in Henan Children’s Hospital and the Third Affiliated Hospital of Zhengzhou University. Among them, CV-A6 was the most important pathogen causing HFMD (652/2850, 22.88%). We found that the D3 subtype gradually appeared from 2011 onward, and all CV-A6 virus strains belonged to the D3 subtype in 2019. We analyzed the molecular evolutionary features of CV-A6 using Bayesian phylogeny and found that the tMRCA of D3 subtype of CV-A6 in China was inferred to be 2005 (95% HPD 2002‐2007), suggesting that the D3 subtype had been circulating in China for many years. In addition, the strain isolated in 2023 had mutations at several amino acid sites compared to the original strain. Some mutations in the evolution of EVs are important for their reproduction, pathogenesis, and transmission [40-42]. These findings can provide ideas for the research of the pathogenic mechanism and allow researchers to look for ways to inhibit viral replication or transmission of new targets.

We note that there has been a significant increase in HFMD outbreaks in Henan Province during the summer months, with peak infections occurring between June and July. This aligns with the epidemic seasons observed in countries such as Thailand and India [20,43]. The specific cause for this has not been clarified. However, climate and geographical location are thought to contribute to the seasonal patterns of HFMD to some degree. High temperature and high humidity have been linked to an increased incidence of HFMD. Records also indicate a correlation between atmospheric pressure, wind speed, and the occurrence of HFMD [44]. Previous epidemiological studies on CV-A6 have revealed a transition from the D2 to D3 branch of CV-A6 evolution occurring in China and France [45,46]. Since 2010, there have been large-scale HFMD outbreaks in several countries, including Brazil [47], China [48], India [49], Japan [50], and Vietnam [51], which have been attributed to CV-A6 D3 strains. Our study also indicates that these CV-A6 D3 strains share closer phylogenetic relationships with viruses that circulated in Thailand, France, Japan, and Vietnam from 2008 to 2021, suggesting the presence of a virus spreading over long distances. Analyzing the phylogenetic tree of the complete VP1 sequence, we found evidence of coevolution between CV-A6 strains prevalent in Henan and CV-A6 strains sourced from other parts of China. This indicates that there may be multiple transmission chains for the strains studied. Given the vast topography and climatic diversity across China, the transmission and replication capacity of CV-A6 may vary by region. It is also worth noting that CV-A6’s subgenotype D3 may be more transmissible, infectious, and virulent, which could potentially explain the sustained international circulation of CV-A6. From the 313 CV-A6 strains included in this study, we constructed a molecular clock evolutionary tree for the VP1 gene. On the basis of previous studies, this study divided CV-A6 into 4 subtypes (A-D), of which D was divided into D1, D2, and D3 subtypes, and described the common ancestor time of each subtype in detail by Bayesian analysis method, which was missing in previous studies [52]. Among them, the first isolation of the D3 subtype of CV-A6 in China occurred in 2011, and the tMRCA was inferred to be 2005 (95% HPD 2002‐2007), suggesting that D3 experienced frequent transmission in China during the 14 years of the epidemic. In addition, the tMRCA of the 2023 isolates was 2019, suggesting that the CV-A6 D3 subtype had been secretly circulating in China for many years since the occurrence of this strain in 2023. This also means that the virus is in the process of continuing to evolve and could lead to large outbreaks in immunity-naive populations. Here, CV-A6 was seen to diverge into 4 evolutionary branches, with the CV-A6 strains situated in the D branch showing more active evolution. This analysis of the evolutionary source implies the importance of bolstering monitoring efforts for strains in the D branch. These strains not only comprise the Henan strains but also include CV-A6 isolates from regions neighboring mainland China. These strains have notably evolved continuously over a relatively short period since 1990, spreading across other regions and forming numerous chains of transmission. The molecular clock evolutionary tree of CV-A6 gives rise to a hypothesis that during the evolutionary genetic process, there were 3 major sources of global CV-A6 transmission: Vietnam; Japan; and various provinces of China, such as Henan, Shandong, Yunnan, etc. Currently, CV-A6 endemic regions are predominantly found in China and its neighboring countries. Mutation and recombination are common processes in EVs, driving significant genotypic and phenotypic variability [53]. These genetic alterations, which can result in antigenic shifts, could potentially spark outbreaks. Nonsynonymous mutations in VP1, leading to changes in amino acids, are frequently found among EVs. Our study has found that the average rates of VP1 nucleotide substitutions fall between 3.8466×10^−3^ and 5.3796×10^−3^ substitutions per site per year in CV-A6, which is consistent with the evolutionary rate estimated from expanded CV-A6 surveys (>300 VP1 sequences) and rates estimated previously [54]. It is imperative to note that genetic alterations at crucial sites in VP1 could give rise to immune-escape mutants. These mutants could impact the efficacy of vaccines as they allow the virus to circumvent immune defenses, possibly leading to poorer control over the disease in populations. Amino acid variants A5T, V30A, S137N, and I242V were also present in the CV-A6 epidemic strain that caused HFMD in Vietnam from 2011 to 2015 [19]. Furthermore, the structure of 137N is a bend, and the substitution from S to N at the 137th position will introduce a new N-glycosylation site [55]; however, the function of this locus needs to be further investigated. The CV-A6 may be mutating toward increased pathogenicity, which may be one of the reasons for the increased prevalence of CV-A6 in Zhengzhou city. Besides, it has been suggested that amino acid mutation sites (96-98, 102, 106, 151, 160, 165, 174, and 216) of the CV-A6 strain may be associated with major neutralizing antigenic epitopes [56]. The amino acid mutation sites (N10S, Q98L, S194T, F261L, and S279T) in this study are also consistent with the epidemiology reported by laboratories in the Philippines [18]. Moreover, we identified previously unreported variants at loci 8, 14, and 32 and other locations in this study, which may be the main drivers of the variants in the VP1 gene of CV-A6. Some mutations in the evolution of EVs are important for their reproduction, pathogenesis, and transmission [40-42]. Furthermore, the emergence of new genotypes or subtypes, intragenotype and intergenotype substitutions, and the introduction of new variants arising from spontaneous mutations and genetic recombination all pose challenges for vaccine development, selection, and vaccination policies. There is a need for laboratory-based surveillance and epidemiological surveillance for genetic change and evolution studies.

A number of important limitations need to be considered regarding these findings. First, as with any common self-limited disease, HFMD surveillance captures only the tip of the clinical iceberg, and most cases go undetected because they are asymptomatic, or because patients do not seek formal care or are not diagnosed and reported. Second, because of the lack of real-time and effective pathogen typing in some areas, the typing of atypical HFMD was insufficient. We may have underestimated the actual depth of harm caused by HFMD. We cannot rule out this monitoring bias. Finally, due to geographical restrictions, this study only selected cases from 2 representative hospitals in Henan Province for virus isolation, with a small sample size. Follow-up studies can further increase the sample size analysis and fully understand the epidemic trend of HFMD.

In this study, we used 34 HFMD-associated CV-A6 strains isolated from Henan Children’s Hospital and The Third Affiliated Hospital of Zhengzhou University, significantly enriching the global CV-A6 VP1 sequence database. We explored the molecular epidemic characteristics of CV-A6 in mainland China and around the world, and we predicted the amino acid mutation sites associated with CV-A6, which deepened our understanding of CV-A6 and provided valuable information for the molecular epidemiology of CV-A6. This not only enhanced our understanding of CV-A6 but also provided valuable insights into its molecular epidemiology. As the pathogen spectrum of HFMD grows more complex, the proportion of previously dominant strains, namely CV-A16 and EV-A71, have decreased. At the same time, there were some rare, easily ignored, and vulnerable epidemic serotypes of EVs that are cocirculating. In light of these findings, a variety of emerging technologies should be used to improve sample detection and enhance surveillance and reporting of other non–EV-A71 and non–CV-A6 EVs. This will enable a more comprehensive understanding of global HFMD epidemiologic trends and provide stronger options for managing and reducing future outbreaks.

## Supplementary material

10.2196/59604 Multimedia Appendix 1 Coxsackievirus A6 sequence numbers for phylogenetic evolutionary analysis.

10.2196/59604 Multimedia Appendix 2 Coxsackievirus A6 VP1 gene sequence numbers for phylogenetic and phylodynamic analyses.

10.2196/59604 Multimedia Appendix 3 Maximum clade credibility tree depicted in FigTree.

## Abbreviations

CV: coxsackievirus
EV: enterovirus
HFMD: hand, foot, and mouth disease
HPD: highest posterior density
MCC: maximum clade credibility
MCMC: Markov chain Monte Carlo
ML: maximum likelihood
tMRCA: time of the most recent common ancestor
